# The application of paravertebral block for open cholecystectomy in a high-risk patient with an implantable cardioverter-defibrillator: a case report

**DOI:** 10.3325/cmj.2025.66.227

**Published:** 2025-06

**Authors:** Miroslav Župčić, Katarina Tomulić Brusich, Tin Nadarević, Sandra Graf Župčić, Viktor Duzel, Gzim Redžepi

**Affiliations:** 1 Department of Anesthesiology, Intensive Care and Pain Medicine, Rijeka University Hospital Center, Rijeka, Croatia; 2 Department of Clinical Sciences II, Faculty of Health Studies, University of Rijeka, Rijeka, Croatia; 3 Department of Anesthesiology, Resuscitation, Emergency and Intensive Care Medicine, Faculty of Medicine, University of Rijeka, Rijeka, Croatia; 4 Department of Radiology, Rijeka University Hospital Center, Rijeka, Croatia; 5 Department of Radiology, Faculty of Medicine, University of Rijeka, Rijeka, Croatia; 6 Department of Neurology, Rijeka University Hospital Center, Rijeka, Croatia; 7 Faculty of Biotechnology and Drug Development, University of Rijeka, Rijeka, Croatia; 8 Northern Hospital Epping, Melbourne, Victoria, Australia; 9 Special Hospital Primamed, Zagreb, Croatia

We present a case of a 57-year-old male patient (American Society of Anesthesiologists status IV) undergoing open cholecystectomy under unilateral thoracic paravertebral block (TPVB) and sedation. The patient had severe heart failure, a reduced ejection fraction of approximately 16%, and an implanted subcutaneous implantable cardioverter-defibrillator. Using ultrasound, we identified the thoracic (Th) paravertebral spaces on the right side at four levels (from Th6 to Th9) and administered 3.5 mL of 0.5% levobupivacaine per level, for a total of 14 mL. Twenty minutes after TPVB application, we confirmed sensory blockade from the Th5 to Th10 dermatomes. Ten minutes into surgery, during liver capsule retraction, the patient experienced some pain (5/10 on the visual analogue scale, VAS). The pain was successfully treated with rescue analgesia of 10 µg of intravenous (IV) sufentanil and a sedation dose of 50 mg IV propofol. The surgery lasted 45 minutes and was completed uneventfully. For continued intraoperative sedation, we used 10 mg/h remimazolam, maintaining hemodynamic stability. Nine hours after surgery, the patient reported a VAS pain score of 5 and received 75 mg of IV diclofenac sodium, with no further analgesia required. The patient was discharged on postoperative day six. In conclusion, the application of TPVB combined with remimazolam sedation could present a feasible anesthetic and analgesic technique for open cholecystectomy in high-risk cardiac patients.

Open cholecystectomy is typically performed under general anesthesia with endotracheal intubation; however, high thoracic epidural or spinal anesthesia can also be used (1,2). Neuraxial anesthesia provides bilateral sympathetic blockade and symmetrical anesthesia but carries risks such as epidural hematoma, nerve damage, and hemodynamic instability, particularly in patients with severe cardiorespiratory disease (1,3). Unilateral thoracic paravertebral block (TPVB) may mitigate these risks while ensuring adequate anesthesia with fewer side effects (4-7). As the patient was very high-risk, the anesthetic technique described is rarely used due to the required expertise and overall risk profile, so it is useful to report on as many of these cases as possible.

## Case report

**Figure 1:**
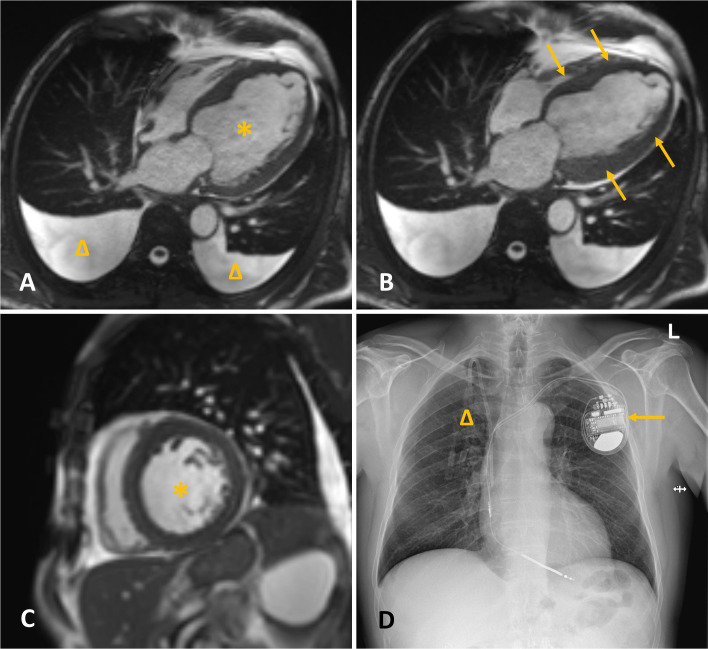
(**A**) Cardiac magnetic resonance imaging (CMR), steady-state free precession (SSFP) cine sequence in end-diastole (ED), 4-chamber view. This image shows an enlarged left ventricle (*) (left ventricular end-diastolic diameter 73 mm, left ventricular end-diastolic volume/body surface area 197 mL/m^2^ [reference values 47-92 mL/m^2]^) and bilateral pleural effusion (Δ). (**B**) CMR, SSFP cine sequence in end-systole (ES), 4-chamber view. In comparison with (**B**), the entire left ventricular myocardium shows severe global hypokinesia (arrows), which results in severely reduced ejection fraction (volumetric analysis showed left ventricular ejection fraction of 16%). (**C**) CMR, SSFP cine sequence in ED, short-axis view. In this image, a dilatation of the left ventricular cavity is seen (*), compared with the right ventricle. (**D**) Chest x-ray in an erect position, postero-anterior projection. A two-chamber cardiac resynchronization therapy device (arrow) and dialysis catheter (Δ) are shown, both in an adequate position. The cardiovascular silhouette is enlarged, lung fields show normal transparency and no pleural effusion.

A 57-year-old patient with American Society of Anesthesiologists (ASA) status IV was scheduled for open cholecystectomy at the Department of Anesthesiology, Intensive Care and Pain Medicine, Rijeka University Hospital Center, due to cholelithiasis and previous gallstone pancreatitis. His comorbidities included ischemic heart disease, arterial hypertension, left bundle branch block, chronic heart failure with a severely reduced ejection fraction of ~ 16%, left heart enlargement, and bilateral pleural effusions, confirmed by cardiac magnetic resonance imaging (MRI) (Figure 1A-C). He also had symptomatic paroxysmal atrial fibrillation with an *in situ* subcutaneous implantable cardioverter-defibrillator (Figure 1D), type 2 diabetes, secondary anemia, right-sided hemiparesis, secondary hyperparathyroidism, and chronic renal failure requiring intermittent hemodialysis. Given these conditions, the patient was at high risk for perioperative cardiac complications, which necessitated an alternative anesthetic approach (8).

Upon arrival to the preoperative holding bay, standard monitoring, including noninvasive blood pressure monitoring, peripheral pulse oximetry (SpO_2_), and electrocardiogram, was applied, and a peripheral intravenous cannula was placed. Following skin infiltration with 2% lidocaine (Lidocaine®, Belupo, Croatia), an ultrasound-guided left radial arterial cannula was inserted for invasive blood pressure (IBP) monitoring.

Under aseptic conditions, using an 8-12 Hz linear ultrasound probe (GE Healthcare, Wauwatosa, WI, USA), the spinous process was identified, and the thoracic (Th) paravertebral spaces were located 2.5 cm lateral to the spinous processes on the right side at a depth of 3.5 cm. The skin and subcutaneous tissue were infiltrated with 2% lidocaine (1 mL per level), followed by the administration of 14 mL (3.5 mL per level from Th6 to Th9) of 0.5% levobupivacaine (Chirocaine®, Abbott, Ireland) with intermittent aspiration.

The patient was then positioned supine, and the vital signs were recorded: IBP, 120/79 mm Hg; heart rate (HR), 67 beats/min; and SpO_2_, 94%. TPVB was assessed with pinprick and temperature tests, confirming blockade from Th5 to Th10 dermatomes on the right side. Sensory blockade was achieved within 20 minutes, and the patient was transferred to the operating theater.

Intraoperatively, sedation was provided with remimazolam (Byfavo®, Paion, Germany) 5 mg IV and maintained at 10 mg/h via a perfusion pump (B. Braun's Perfusor®, Melsungen AG, Germany). Monitoring included IBP, SpO_2_, HR, and the visual analogue scale (VAS; 0 = no pain; 10 = maximum pain), recorded every five minutes.

Approximately ten minutes into the operation, the patient reported pain (VAS 5) during liver capsule retraction, which was treated with 10 µg IV sufentanil (Laboratoire Renaudin, Itxassou, France) and a 50 mg IV propofol bolus (Fresenius, Bad Homburg vor der Höhe, Germany). Five minutes later, IBP dropped to 85/60 mm Hg, and HR decreased to 58 beats/min. The patient received 5 mg IV ephedrine hydrochloride (Biotika, Prague, Czech Republic), restoring IBP to 105/60 mm Hg and HR to 63 beats/min. No further hemodynamic instability occurred.

The patient breathed spontaneously at 14 to 18 breaths per minute with 4 L/min oxygen via a non-rebreathing mask. The 45-minute procedure required no additional analgesia or sedation.

Postoperatively, the patient was monitored in the post-anesthesia care unit for one hour before transfer to the ward. Vital signs and pain scores were assessed every three hours. Nine hours post-surgery (10.5 hours after block application), the patient reported a VAS score of 5/10 and received 75 mg IV diclofenac sodium (Voltaren, Pliva, Zagreb, Croatia), requiring no further analgesia.

During recovery, the patient showed significant improvement, receiving antibiotic therapy and hemodialysis. On postoperative day six, he was discharged home with stable parameters and no complications. Key perioperative events and investigations are summarized in Table 1.

**Table 1:** The timeline of diagnostic tests and interventions*

Date	Diagnostic tests and interventions
August 3, 2023	CVI with right-sided hemiparesis
August 4, 2023	Renal replacement therapy with hemodialysis
September 9, 2023	Acute gallstone pancreatitis
October 7, 2023	Cardiac MRI
February 9, 2024	Implantation of a S- ICD
March 8, 2024	Open cholecystectomy surgery
March 14, 2024	Discharge home from hospital

*Abbreviations: CVI – cerebrovascular insult; MRI – magnetic resonance imaging; S-ICD – subcutaneous implantable cardioverter-defibrillator.

## Discussion

Patients undergoing open cholecystectomy who are at high risk for general or neuraxial anesthesia due to severe cardiac and respiratory impairment may benefit from unilateral TPVB (1-7). Literature on this technique remains limited (6,7).

Consistent with Serpetinisa et al (6), we administered a long-acting local anesthetic (0.5% levobupivacaine) across multiple thoracic levels to improve distribution and minimize adverse effects (5,9,10). We applied 3.5 mL per level at four levels to optimize anesthetic, analgesic, and hemodynamic outcomes.

In contrast, Beyaz et al (7) used a single Th7 injection of 20 mL 0.5% levobupivacaine in two ASA III patients with a nerve stimulator, reporting significant hypotension (65/44 mm Hg) in one case. In our patient, ultrasound guidance improved precision and reduced side effects (5,10). The brief hypotensive episode likely resulted from sufentanil and propofol administration for rescue analgesia and sedation during liver capsule retraction.

To minimize hemodynamic instability, we opted for remimazolam over propofol for continued intraoperative sedation (11). Although a perineural catheter could have provided prolonged postoperative analgesia, the inability to monitor invasive blood pressure on the ward led us to forgo this option for safety reasons.

In conclusion, TPVB with reduced-dose local anesthetic, combined with remimazolam sedation, appears to be a safe and effective anesthetic strategy for open cholecystectomy in high-risk cardiac patients. This technique provides effective anesthesia and analgesia while minimizing hemodynamic instability and respiratory compromise, which makes it a viable alternative in select cases.
